# Mixed Aetiology of Diarrhoea in Infants Attending Clinics in the North-West Province of South Africa: Potential for Sub-Optimal Treatment

**DOI:** 10.3390/pathogens9030198

**Published:** 2020-03-06

**Authors:** Martina O. Chukwu, Akebe Luther King Abia, Eunice Ubomba-Jaswa, John Barr Dewar, C.L. Obi

**Affiliations:** 1 Department of Life Sciences, College of Agriculture and Environmental Sciences, University of South Africa, Corner Christiaan De wet and Pioneer Avenue, Florida Park, Roodepoort 1724, Gauteng, South Africa; dewarj@unisa.ac.za; 2 Antimicrobial Research Unit, College of Health Sciences, University of KwaZulu-Natal, Private Bag X54001, Durban 4000, South Africa; lutherkinga@yahoo.fr; 3 Department of Biotechnology, University of Johannesburg, 37 Nind Street, Doornfontein 2094, Gauteng, South Africa; euniceubombajaswa@yahoo.com; 4 Water Research Commission, Lynnwood Bridge Office Park, Bloukrans Building, 4 Daventry Street, Lynnwood Manor, Pretoria 0081, South Africa; 5 School of Science and Technology, Sefako Makgatho Health Science University, Ga-Rankuwa 0208, South Africa; c355251@gmail.com

**Keywords:** bacteria, mix-aetiology, *Campylobacter*, *Arcobacter*, diarrhoeagenic *E. coli*, virulence genes, diarrhoea, viruses

## Abstract

Routine diagnostic methods for the aetiologic agents of diarrhoea in most developing countries are usually not sensitive enough, leading to under-diagnosis. Thus, this study investigated possible mixed diarrhoeal aetiology by using cultures and real-time polymerase chain reactions (PCR) in children younger than four years old in the Northwest Province, South Africa. In total, 505 stool samples were collected from symptomatic and asymptomatic children who were attending three clinics and the Brits hospital in Madibeng District, between September 2016 and December 2017. Rotavirus, norovirus, *Campylobacter*, *Arcobacter*, and diarrhoeagenic *Escherichia coli* (DEC) were targeted. *Campylobacter* spp. (24.6%), *Arcobacter* (15.8%) and DEC (19.6%) were detected using PCR; only *Campylobacter* spp. (29.7%) and DEC (26.9%) were detected through the culture. *Campylobacter jejuni* (36%), *Campylobacter coli* (28%), *Campylobacter upsalensis* (12%), and *Arcobacter butzleri* (15.8%) were the only spp. of *Campylobacter* and *Arcobacter* identified. The *eaeA* gene (31.4%) of enteropathogenic *E. coli*/enterohaemorrhagic *E. coli* (EPEC/EHEC) was the most prevalent DEC virulence gene (VG) identified. *Rotavirus* and *norovirus* were detected at 23.4% and 20%, respectively. Mixed viral aetiology (7.3%) and the co-infection of *A. butzleri* and *Campylobacter* (49%) were recorded. A mixed bacterial-viral aetiology was observed in 0.6% of the specimens. Sensitive diagnostic procedures like PCR should be considered to provide the best treatment to children experiencing diarrhoea.

## 1. Introduction

Diarrhoea is the most common clinical manifestation of gastrointestinal diseases and is defined as the frequent discharge of unformed, loose, or watery stool, usually three or more times in 24 h [1,2]. Diarrhoea is associated with a high morbidity and mortality and is among the leading causes of the global year of life lost (YLL) in people of all ages [3]. It is a significant cause of death in children younger than five years in developing countries [3,4]. In Africa, diarrhoeal diseases are responsible for 26% of childhood deaths [3,4]. These diseases are usually caused by various enteric pathogens such as rotavirus, norovirus, sapovirus, *Escherichia coli*, *Campylobacter* spp., *Shigella* spp., *Aeromonas* spp., *Plesiomonas* spp., *Arcobacter* spp., and *Salmonella* spp. [1,5,6].

Of all the acute infections associated with these enteric pathogens, rotavirus diarrhoea is the most frequently reported [1]. It has been reported to account for the global deaths of approximately 453,000 children younger than five years, with over 90% of these deaths occurring in Africa and Asia [7]. Before the introduction of the rotavirus vaccine, rotavirus was responsible for one in four cases of hospital admission due to diarrhoea in South Africa [8]. However, since the introduction of the rotavirus vaccine in many countries, rotavirus-associated diarrhoea has reduced [9], and diarrhoea due to other enteropathogens such as noroviruses, *Campylobacter* spp., *Arcobacter* spp., and diarrhoeagenic *E. coli* (DEC) has shown a relative increase in incidence [6,10,11]. 

Noroviruses are known causes of viral gastroenteritis in people of all age groups, especially in immunocompromised patients, the elderly [12], and in younger children [10]. These small single-stranded, positive-sense RNA viruses with a genome of around 7500 nucleotides [13] are responsible for 677 million cases of diarrhoea globally [11]. They are classified into six genogroups (GI–GVI) and 36 genotypes [14] and are often associated with gastrointestinal outbreaks in semi-closed environments such as nursery schools, hospitals and ship cruises [12]. 

Diarrhoeagenic *E. coli* are considered the leading agents of bacterial gastroenteritis and foodborne pathogens that cause acute and chronic paediatric diarrhoea [15,16,17,18]. *E. coli* typically colonises the gastrointestinal tract of humans within hours of birth [19] but used to be considered harmless resident of the gastrointestinal tract and used as indicators of faecal contamination. However, recent research advances have shown that some strains have become pathogenic by acquiring virulence factors that enable them to attach to human cells and initiate infections [20]. Globally, DEC strains are the cause of most paediatric diarrhoea, foodborne infections, and traveller’s diarrhoea [11,17]. Several DEC pathotypes have been identified and grouped according to the severity and symptoms of diseases they produce and their associated virulence genes [21]. Such pathotypes include enterohaemorrhagic *E. coli* (EHEC), enteroaggregative *E. coli* (EAEC), enteropathogenic *E. coli* (EPEC), and enteroinvasive *E. coli* (EIEC) [1] (Cooper et al., 2014). The EHEC strains are also known as Shiga-toxin-producing *E. coli* (STEC) and are the frequent cause of bloody diarrhoea and haemolytic uremic syndrome [22]. The EIEC members are the frequent cause of watery diarrhoea and occasionally dysentery in both children and adults [23]. The EPEC strains mainly cause diarrhoea in children, particularly under poor hygienic conditions, as well as in animals. [24,25]. The ETEC pathotype is a frequent cause of persistent diarrhoea in developing countries [18,26].

*Campylobacter* and *Arcobacter* are responsible for over 18% of diarrhoea cases in the developing world, with most cases reported in children younger than two years old. They are gram-negative, non-spore forming, zoonotic bacteria, and they are members of the Campylobacteraceae family. They are cable of causing infection in humans, especially young children, at a relatively low infectious dose [27]. They attach to the human host after exposure to contaminated farm animals, water, or food [15,28]. They are major agents of foodborne diseases and precursors to more severe illness, including immune-reactive complications such as Guillain–Barre Syndrome (GBS) and Miller Fisher Syndrome (MFS), a chronic and potentially fatal form of paralysis [29]. Most human campylobacteriosis cases are caused by *Campylobacter jejuni*, *Campylobacter coli*, and *Campylobacter upsalensis*, with over 90% of such cases being caused by *C. jejuni*; most arcobacteriosis cases are due to *Arcobacter skirrow*, *Arcobacter butzleri* and *Arcobacter cryaerophilus* [30]. In most symptomatic cases, campylobacteriosis manifests as mild and self-limiting gastroenteritis characterised by one-to-three days of fever, vomiting, headache, watery or bloody diarrhoea [31]. 

Routine laboratory identification of these aetiological agents usually involves culture and biochemical tests. Though culture methods are considered the gold standard, they are time-consuming and may not be as sufficiently sensitive as polymerase chain reaction (PCR) assays [32]. Campylobacteraceae are fastidious microorganisms, and their laboratory identification is often challenging because it involves the use of selective and differential culture media, as well as biochemical and serological tests, which may not differentiate the species [33]. 

The culture of DEC is also problematic since even selective media and most biochemical assays do not distinguish DEC from the non-pathogenic strains. Additionally, serotyping does not always correlate with pathogenicity. However, several virulence factors and DNA sequences associated with DEC have been identified and can only be determined by detecting the presence of genes coding for specific virulence factors that are absent in non-pathogenic strains [19]. These genes have been characterised and can be identified by PCR [34]. Efforts to cultivate noroviruses and rotaviruses in cell culture are not routine, although electron microscopic characterisation and serologic responses are used to identify these viruses; clear and specific identification can only be achieved through PCR [13,35].

In many developing countries, the aetiology associated with diarrhoeal diseases are often unknown [4]. This is mainly due to a failure to isolate the causative agents in the laboratory or isolating one agent and leaving the other; both diagnostic factors affect the management and treatment of the disease. [32,36]. To effectively control diarrhoeal diseases in any community, an accurate understanding of the relative aetiologic agents and early detection of these agents using appropriate diagnostic tools is necessary. Therefore, the goal of this study was to use culture-based and real-time PCR assays to detect rotavirus, norovirus, DEC, *Arcobacter* spp., and *Campylobacter* spp. to determine their involvement in the aetiology of gastroenteritis in paediatric patients in the North-West Province, South Africa.

## 2. Results

### 2.1. Distribution of Participants According to Age, Mode of Feeding and Presence/Absence of Diarrhoea

A total of 505 stool specimens were collected from children ≤4 years. Of the 505 participants, 350 had diarrhoea or symptoms of gastroenteritis at the time of sample collection, and 155 were healthy controls with no episode of diarrhoea or gastroenteritis on the day sample was collected. The number of children recruited in each category is shown in Table 1. Children under the age of one were the most recruited, and they make up for more than 50% of the gastrointestinal cases. 

### 2.2. The Overall Distribution of Aetiologic Agents among the Study Population

Of the 505 samples analysed, the rate of recovery of *Campylobacter* was 54.3% (274/505), *E. coli* 46.5% (235/505), *Arcobacter* 15.8% (80/505), rotavirus 23.4% (118/505), and norovirus 20% (101/505). Of the 274 identified *Campylobacter* positive samples, 150 (29.7%) were identified using the culture method and 124 (24.6%) were identified by real-time PCR. Out of the 235 DEC identified, 99 (19.6%) were recovered from whole stool sample using real-time PCR alone and 136 (26.9%) were from pure culture. Rotavirus, norovirus and *Arcobacter* spp. were only identified using real-time PCR.

### 2.3. Prevalence of Aetiologic Agents in Symptomatic Participants

All studied microbial species were more associated with diarrhoea (Table 2). None of the viruses was isolated from participants with bloody diarrhoea.

### 2.4. Prevalence of Arcobacter and Campylobacter spp. in the Study

The amplification of the *Arcobacter* using PCR allowed for the identification of *Arcobacter* in 15.8% of the samples. *Arcobacter butzleri* was identified more from female participants 52.5% (42/80) than male participants 47.5% (38/80). *Arcobacter cryearophilus* and *Arcobacter skirrowii* were tested but were not found in the study population. According to the method of feeding, *A. butzleri* was more prevalent among babies who received a mixed diet 52/80 (65%) compared to those on an exclusive breast-feeding diet 28/80 (35%). Based on clinical presentation, *A. butzleri* was significantly associated with infants with diarrhoea 56/80 (70%) compared to those without 30% (24/80) (*p* = 0.044). Regarding age, *A. butzleri* was only isolated in children within the 0–24 months age range.

*Campylobacter*, on the other hand, was identified in 54.3% of the samples. Of these, *C. jejuni* were the most detected (39.4%; n = 108), followed by *C. coli* (32.4%; n = 89) and *C. upsaliensis* (14.5%; n = 40). Additionally, combinations of *Campylobacter* spp. were detected in 12% (n = 33) of the samples. The majority of *Campylobacter*-positive samples, 78.8% (216/274), were from children in the age group of 0–12 months. 

*Campylobacter* infection was more common in infants with diarrhoea (74%; 205/274) compared to infants without diarrhoea (25%; 69/274) (*p* = 0.044), and 16% (45/274) were found in samples with bloody diarrhoea. Additionally, *C. jejuni* was more associated (*p* < 0.05) with diarrhoeal cases (64.8%; 70/108) compared to non-diarrhoeal cases (26.8%; 29/108).

Similarly, *Campylobacter* was more detected in babies on a mixed feeding diet (65.7%; 180/274) than those on an exclusive breastfeeding diet (34.3%; 94/274). The rate of *C. jejuni* infection was significantly higher (64.8%; 70/108) (*p* = 0.04) in children on a mixed feeding diet than in those on an exclusive breastfeeding diet (26.8%; 29/108). Generally, *Campylobacter* infection was detected more in male babies (42.3%; 116/274) than in female babies (31.8%; 87/274) where infection with *C. coli* (64.9%) and *C. upsaliensis* (55.5%) showed a higher prevalence among the male participants compared to *C. jejuni* (51.5%). 

### 2.5. Prevalence of DEC Virulence-Associated Genes

**Figure 1:**
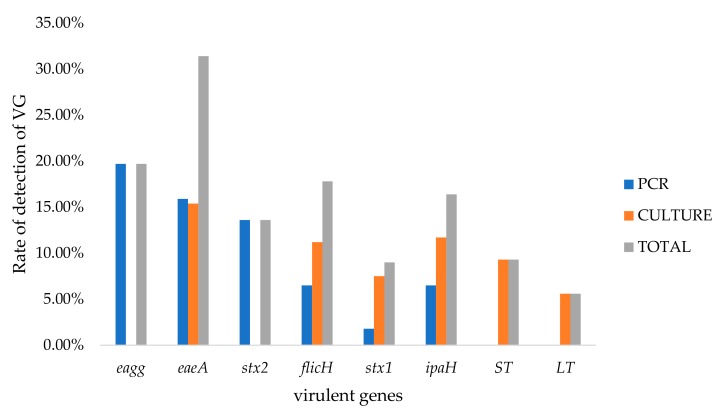
Rate of expression of diarrhoeagenic *Escherichia coli* (DEC)-associated virulence genes in whole stool samples and pure colonies.

Of all the samples that tested positive for *E. coli* (PCR amplification of the *mdh* gene), DEC virulence genes were detected in 88 of these samples. Similarly, from the 136 pure *E. coli* isolates, PCR identified DEC-associated virulence genes (VGs) in 125 of them. The rate of detection of virulence genes from pure isolates and whole samples is shown in Figure 1. 

The *ST* and *LT* genes were not amplified in the DNA isolated from the whole samples, and the *stx2* and *eagg* genes were not detected in the pure isolates. The *eaeA* gene was the most commonly detected DEC-associated VG from both the whole sample and pure culture, while *stx1* was the least detected gene. A combination of the *stx2* and *flicH7* genes was detected in three samples, while the *eaeA* and *stx2* genes were observed in 12 samples. The co-detection of the *eagg, stx1*, *flicH7*, and *eaeA* genes was also recorded.

Additionally, the various DEC VGs were not equally distributed in the children with respect to the presence or absence of symptoms, as well as their mode of feeding (Table 3).

### 2.6. Prevalence of Rotavirus and Norovirus

The prevalence of rotavirus infection in the children recruited into this study was 23.4% (118/505). The age of children affected with rotavirus was restricted to those between the ages of 0 and 25 months. The majority of rotavirus infection, 82.2% (97/118), occurred in children in the 0–12 months age group. Of the 118 children who tested positive for rotavirus, 55% (n = 65) were females and 44.9% (n = 53) males. Episodes of vomiting were reported in 29.7% (n = 35) of the 118 children, while 39.8% (n = 47) of the babies showed symptoms of fever. Of these, 62.7% (n = 74) were on a mixed-feeding diet, 69.5% (n = 82) experienced diarrhoea, and 57.6% (n = 68) had received the rotavirus vaccine.

On the other hand, norovirus infection was recorded in 20% (n = 101) of the children participating in the study. The age of the children affected was between 0 and 30 months. The highest rate of infection was noted in children in the age group of 0–12 months (84.2%; n = 85). As opposed to the gender distribution regarding rotavirus infection, more male children were infected with norovirus (53.5%; n = 54), and the gender difference was statistically significant (*p* = 0.045). Of the 101 detected cases of norovirus infection, 76.2% (n = 77) were from symptomatic cases, while 23.8% (n = 24) were from asymptomatic cases. Vomiting and fever were reported in 39.6% (n = 40) and 54.5% (n = 55) of the norovirus-positive cases, respectively.

### 2.7. Prevalence of Mixed Aetiology

The mixed aetiology of *A. butzleri* and *Campylobacter* spp. was found in 9.7% (n = 49) of the samples, while the mixed infection of all the tested bacteria was observed in 1.2% (n = 6) of the samples. Of these, three babies were under the age of six months, all six children were on a mixed feeding diet, and four of the six children (66.6%) had bloody diarrhoea.

Of the children infected with rotavirus (n = 118) and norovirus (n = 101), 16.9% (n = 37) were confirmed to have viral co-infection and were mainly seen in the diarrhoeal cases (75.8%; n = 28). These children were in the age group of 0–24 months. Episodes of fever were recorded in 48.6% (18/37) of the co-infected children, most of whom (70.3%; n = 26) were on a mixed-feeding diet and 51.4% (n = 19) had received the rotavirus vaccine.

Bacterial and viral co-infection was detected in three children who were aged three, four and 18 months, and two of those presented with bloody diarrhoea, fever, and vomiting. One of these three children had received two doses of the rotavirus vaccine, and the other had not been vaccinated against rotavirus. 

## 3. Discussion

Despite advances in the understanding of pathogen transmission patterns, diarrhoeal diseases remain the leading causes of morbidity worldwide. A vast number of pathogens can cause diarrheal diseases, but only a few account for the majority of the disease burden worldwide [1]. This study was undertaken to determine the aetiologic agents responsible for diarrhoea in the North-West Province of South Africa by analysing faecal samples of symptomatic and asymptomatic children under the age of five years. The study focused on *Campylobacter* species (*C. coli, C*. *jejuni and C. upsalensis*), DEC virulence-associated genes (*ST*, *LT*, *stx1*, *stx2*, *flicH*, *ipaH*, *eaeA* and *eagg*), *A. butzleri*, rotavirus, and norovirus. 

Due to the fastidious nature of and special growth requirements for Campylobacteraceae, the current study used both conventional culture methods and real-time PCR assays to detect bacterial agents. Using only the culture method, *Campylobacter* spp. (29.7%) and *E. coli* (26.9%) were identified in the collected stool samples. The negative stool samples were further analysed using the *16S rRNA* gene for *Campylobacter* identification, *23S rDNA* gene for *Arcobacter* spp and *mdh* gene for *E. coli* identification. This method identified *Campylobacter* in 24.6% (150), *Arcobacter* in 15.8% (80), and *E. coli* in 19.6% from culture-negative samples. These results confirm that, although culture methods are suitable, they are not sufficient to use alone in epidemiological studies, because they might not give a true reflection of the prevalence of the aetiologic agents involved in any given situation. For example, 24.6% of *Campylobacter* spp., 15.8% of *Arcobacter* spp., and 19.6% of *E. coli* would have gone undetected when using only the culture method. These results corroborate the report of Bessede et al. [37], who reported a low sensitivity of culture over PCR. Similarly, Lawson et al. [38] identified 13.1% of *Campylobacter* spp. from faecal specimens that were previously found to be negative when using the culture method [38]. These differences are due to the higher sensitivity of PCR over culture. Based on the identification of DNA, PCR also identifies the presence of free DNA and viable but non-culturable bacteria that would otherwise not be detected when using culture-dependent techniques. Nevertheless, the detection of these isolates suggests the exposure of the participants to these pathogenic microbes.

The detection frequency of all the aetiologic agents identified in this study showed that *Campylobacter* spp. were the major agents of diarrhoea within the studied community, followed by *E. coli*, rotaviruses, noroviruses, and *A. butzleri*, as they were the most isolated from diarrhoeal samples. In agreement with this study, *Campylobacter*, *E. coli*, norovirus, and rotaviruses have been recognised agents of diarrhoea both in community and clinical settings [39,40,41]. The higher detection rate of these aetiologic agents among the symptomatic babies in this study confirms that these agents might have contributed to causing diarrhoea in the studied babies. Higher prevalence of aetiologic agents were noticed more in the samples of babies on mixed feeding diets compared to those on exclusive breastfeeding diets. These results support the previous finding that babies on exclusive breastfeeding diets are less prone to diarrhoea diseases [42,43]. 

### 3.1. Occurrence of Bacterial Agents in the Present Study

#### 3.1.1. Occurrence of Campylobacter

The prevalence of *Campylobacter* reported in the present study is slightly higher compared to the 30.4% and 38.9% previously reported in South Africa [44,45]. This difference might be attributed to the two methods used for isolation. However, it reaffirms the presence of *Campylobacter* infection among children in South Africa and the need to increase surveillance. Three prominent *Campylobacter* spp. were identified, with *C. jejuni* being the dominant species. A higher percentage isolation of *C. jejuni* over other *Campylobacter* spp. has been reported in studies all over the world. In developed countries such as the USA, 75% of *C. jejuni* and 4% *C. coli* have been reported [46]. Similarly, 85% *C. jejuni* and 15% *C. coli* have been documented in South Africa [45]. Infection due to *C. jejuni*, in most cases, can lead to neurological sequalae such as Guillain–Barre syndrome (GBS) [47]. *C. jejuni* has been noted to be the most prominent of all *Campylobacter* species worldwide, and it has been reported to be responsible for most human campylobacteriosis [37]. In the current study, although *C. jejuni* was the dominant species identified in all the samples, a higher prevalence of *C. coli* was observed among children with clinical symptoms compared to the other *Campylobacter* spp. Additionally, the 9.9% of identified *C. upsalensis* was noted more in samples from asymptomatic children. *C. upsalensis* is mostly associated with household pets such as cats; though rarely found in clinical specimens, it was reported to be the cause of multiorgan failure in a patient in Japan [48].

The trend of *Campylobacter* infections according to age observed in this study supports earlier reports that *Campylobacter* infections are prevalent among children under the age of two years in developing countries, and these infections decrease with an increase in the age of the children [49,50]. In studies including age groups, a *Campylobacter* infection is usually high among the children participants. For example, in Peru, a 41.3% prevalence was reported in children [6] and in India, it was found to be associated with 70% of diarrhoeic children [51]. In South Africa, it was reported in 30% of children under the age of two years [45]. The results of all these studies showed that children are more prone to *Campylobacter* infection, and control programs targeting the eradication of infections due to *Campylobacter* should prioritize children and their caregivers, as diarrhoea due to *Campylobacter* has been reported to be associated with a growth shortfall in children [52]. 

#### 3.1.2. Prevalence of Arcobacter

Though the presence of other *Arcobacter* species was investigated in the current study, only *A. butzleri* was detected. However, this specie was the least detected pathogen in our entire study, with a prevalence of 15.8%, and it was only identified through PCR. These results agree with a case report presented by Arguello et al. in which the authors failed to identify causative pathogens from the stool sample of a patient with bacteraemia through the conventional methods used in their laboratory; however, subsequent testing with molecular assays revealed the presence of *A. butzleri* [53]. Though there is no defined mortality or morbidity rate for *A. butzleri*, it has been identified in many studies as an agent of diarrhoea and bacteraemia, particularly in children and immunocompromised individuals [54,55]. Depending on the prevalence of *A. butzleri*, exposure to risk factors within the community, or the method of identification, different percentages of *A. butzleri* have been reported. In a previous study in South Africa, 6.5% of *A. butzleri* was reported [45]; additionally, percentages of 56.7% from diarrhoeic and 45.5% from non-diarrhoeic specimens were reported in Canada [56]. Virulence genes have been reported in *A. butzleri* in several studies [57,58,59]. 

The presence of these virulence genes in *A. butzleri* warranted the international commission of microbiological specification of foods (ICMSF) to categorize *A*. *butzleri* as a serious hazard to humans [59,60].

#### 3.1.3. Prevalence of DEC Pathotypes

The pathogenic strains of *E. coli* are well-known causes of severe diarrhoea outbreaks worldwide [61]. In this study, eight DEC virulence genes representing five *E. coli* pathotypes were analysed. A higher prevalence of these pathotypes was observed in diarrhoeic stool samples compared to non-diarrhoeic ones. The most prevalent gene was the *eaeA* (31.4%), which is common in EHEC and EPEC pathotypes. This gene was also more associated with diarrhoea compared to non-diarrhoea samples. For many years, typical EPEC were considered to be a major agent responsible for diarrhoea in children, particularly in developing countries. However, a few sporadic reports have indicated the association of atypical (aEPEC) with children [62,63,64]. In developing countries, it has also been recognised as an agent of infantile diarrhoea, and it can also be found in non-diarrhoea samples [65,66]. In South Africa, a prevalence of 20% was reported in children with diarrhoea [61], while a prevalence of 19% has been reported in Mozambique [67]. In this study, it was observed that the *eaeA* gene was expressed more from samples of children without fever and vomiting, agreeing with previous a study in Denmark where 44% of infected children did not show any sign or symptom of diarrhoea [68]. Such children could be carriers and transmit the infection to other vulnerable children, especially in resource-poor settings with poor hygiene and sanitation facilities.

Following aEPEC/EHEC, the *eagg* gene of the EAEC pathotype was the second most identified gene (31.4%) in the DEC-positive samples. It was mostly associated with diarrhoea and a mixed feeding diet. Similar to these findings, EAEC was the prominent pathotype detected in a previous study in South Africa; it was found in 57.3% of those studied [61]. Contrary to the present study, it was detected majorly from non-diarrhoea stools. EAEC pathotypes have emerged as enteric pathogens responsible for acute and persistent diarrhoea and may cause malnutrition and growth defects in children [69,70]. These pathotypes are known agents of traveller’s diarrhoea [71,72]. Though they are frequently isolated in asymptomatic cases, reports have shown that even in the absence of diarrhoea, they are associated with intestinal inflammation, which may impact child’s development [73]. In developing countries, the prevalence of EAEC pathotypes among diarrhoea cases ranges from 4.5% to 41% [69,71,74,75]. Studies carried out in Europe and Romania have implicated the EAEC pathotype of *E. coli* as the common bacterial cause of diarrhoea [76,77,78]. 

In addition to the *eagg* gene, the EHEC virulence genes *stx1*, *flicH7*, and *stx2* were expressed in 9%, 17.8%, and 13.6% of those studied, respectively. EHEC pathotypes, also known as Shiga toxin *E. coli* (STEC), are a public health concern because of the severity of their infections. Their primary virulence factor is the Shiga Toxin (stx), although they produce several other virulence genes, with the *stx1* and *stx2* genes being the major ones, used to disrupt host protein synthesis, causing apoptotic cell death [79]. Studies performed in other regions have reported low prevalence rates of the STEC pathotypes, such as in Argentina (6.2%), Mexico (2.5%), Colombia (1.8%), and Brazil (0.5%), while in South Africa, a rate of 3.8% has been observed in non-diarrhoea stools [61]. The expression of stx by *E. coli* is associated with the development of haemolytic-uremic syndrome (HUS) in up to 10% of STEC infections [80,81]. Though HUS can develop in patients of any age, it is a potential life-threatening condition and has been frequently observed in children [81,82]. It is a recognized cause of renal failure and hypertension in children in the UK [83]. 

The *IpaH* gene, which is common to *Shigella* and the EIEC pathotype, was among the least detected genes in this study. It was found in 16.4% of the samples, of which 68.5% were from diarrhoea samples. These results suggest that the EIEC pathotype might be one of the active agents of diarrhoea among the studied population, although to a lower degree. Several studies have reported on the prevalence of the *ipaH* gene [84,85]. EIEC pathotypes are known to present with diarrhoea and dysentery, similar to *Shigella* spp. in humans [19]. 

The ETEC pathotype, produced the *ST* (heat stable) or in combination with *LT* (heat labile) genes, is also a major contributor of diarrhoea burden in both developed and developing countries [1,18]. These genes were expressed in 9.3% (*ST*) and 5.6% (*LT*) in this study and were more associated with clinical symptoms. These results concur with the report of Mclamb et al. [86]. In their study, they investigated the clinical onset and severity of intestinal disorder exhibited by ETEC in humans, and they observed that ETEC was capable of inducing serious clinical symptoms including persistent diarrhoea, loss in weight, fever, intestinal injury [86], and growth impairment [26].

### 3.2. Prevalence of Viral Aetiology 

Viral agents were detected in 36% of the children in the current study. All the rotavirus-infected children were under the age of three years, and 57.6% had received the rotavirus vaccine. The most affected (82.2%) were in the 0–12 months age group. These results correspond with a recent published report on the effectiveness of the rotavirus vaccine in South Africa [7]. Prior to the rotavirus routine immunization programme in South Africa, rotavirus accounted for 35% of all hospital admissions [7]. The current study found a 23.4% prevalence of rotavirus infection, and over 50% of the children had received the rotavirus vaccine. These results supports the fact that the rotavirus vaccine provides protection against rotavirus-associated diarrhoea [7]. Though mortality due to rotavirus has decreased, morbidity seems to be stable, possibly because the available vaccine may not be effective against emerging rotavirus genotypes. Previous studies have reported that, after the introduction of the vaccine in some African countries, genotypes that were not present during the vaccine formulation emerged [87,88,89]. Additionally, during the rotavirus vaccine clinical trial, vaccine efficacy was reported to be low in developing countries compared to developed countries [7].

With the increased use of the rotavirus vaccine, norovirus has been recognised as a leading cause of medically attended diarrhoea in children and adult worldwide. The prevalence of norovirus in many African countries is unknown, with varying percentages of 22% in Botswana [90], 21% in Libya [91] and 18.9% in Kenya [92] being reported. The present study detected norovirus in 20% of the study population. These variations are mostly due to the study duration, population groups, sample size, and inclusion of asymptomatic controls in the studies. In the present study, norovirus was detected only in children younger than two years old; the highest infection (84.2%) was recorded among the children under 12 months of age. These results are in agreement with previous studies in South Africa [93] and Brazil [10], where norovirus infection was frequently detected in children younger than two years old. Though norovirus infections are perceived as self-limiting, infection in children could cause serious symptoms that may require hospitalization. Studies have shown that an infection with norovirus only provides short term immunity, such that pre-existing antibodies will not proffer protection against reinfection [94].

### 3.3. Prevalence of Mixed Aetiology 

A high prevalence of mixed aetiology with different DEC pathotypes and *Campylobacter* spp. was observed in the present study. A mixed aetiology raises the question of whether a single pathogen is responsible for illness or whether several pathogens act in synergy. Some studies have confirmed a mixed aetiology of pathogens in patients. In India, for example, 11.3% of a mixed aetiology of rotavirus and other pathogens was found [63]. In Tanzania, Moyo et al. [95] reported a 20.7% mixed aetiology of DEC among children younger than five years old [95]. Of the 219 children positive for rotavirus or norovirus, 16.9% showed mixed aetiology. In the present study, mixed aetiology was observed both in children with diarrhoea and healthy babies. Other studies have noted that a mixed aetiology of two or more agents and up to five associated pathogens could be found in a host [63]. The present study found that mixed aetiology was more prevalent among children on a mixed feeding diet. This might be a confirmation that gastrointestinal pathogens circulate in the population with ease and that the environment, food, and water may be vehicles of transmission of these agents [96]. It has been reported that the occurrence of a mixed aetiology of infections in a patient is common among individuals living in a community where good sanitation and hygiene is compromised [97]. This ties with the current study, as all the babies that participated were from a rural community; this result could be an indication that environmental factors such as infected stray animals, poor sanitation, and an inconsistent water supply could be some of the reasons for the high mixed aetiology. 

Another reason for the detection of a mixed aetiology of an infection could be the method of identification used. In the current study, the culture method and PCR with DNA extracted directly from the samples were used in parallel, which reduced the error of missing positive samples via culturing alone and biochemical identification. However, it should be noted that although PCR is a highly sensitive method, its complexity may be a limitation for its use in routine diagnostic laboratories. As such, the results of this research are meant to stimulate the need for the development of diagnostic methods that would be inclusive of more aetiologic agents than just the ones currently tested. 

## 4. Materials and Methods 

### 4.1. Specimen Collection

Stool samples were collected from September 2016 to December 2017 from children ≤4 years old, with or without diarrhoea, and attending the Brits District Hospital, Oukasie, Lethabile and Bopang Clinics in the Madibeng District of the North-West Province, South Africa. Diarrhoeal samples were consecutively collected from patients who presented with acute diarrhoea and were not on any antimicrobial therapy, while non-diarrhoeal samples were taken from babies who did not present with any gastrointestinal complaints. A single sample was collected per participant, and participants whose samples had been collected earlier in the study were excluded in order to avoid multiple collections of samples from one participant. Samples were collected in sterile, wide-mouthed, 10 mL bottles with tight-fitting leak-proof lids. These were carefully labelled before being put in a cooler box containing ice packs and transported to the Microbiology Laboratory of the Council for Scientific and Industrial Research (CSIR), Pretoria, within two hours of collection for further processing.

After proper counselling about the study, informed consent was obtained from the parents/guardians attending to the study participants. Detailed personal history, diarrhoeal episode, and associated signs and symptoms were recorded on a questionnaire before collecting the stool samples.

### 4.2. Isolation of Campylobacter and Arcobacter Species

The methods described by Bessede et al. [37] and Shah et al. [98] were employed for isolation of *Campylobacter* and *Arcobacter*, with slight modifications. Briefly, stool samples were enriched with *Campylobacter* growth supplement (ThermoScientific, Johannesburg, South Africa) in a Bolton broth (BB) and incubated at 37 °C for 24 h in a microaerophilic atmosphere (MAE) (CampyGen^TM^, ThermoScientific, Johannesburg, South Africa). *Arcobacter* was isolated in an *Arcobacter* broth (AB) supplemented with CAT (cefoperazone, teicoplanin and amphotericin B; Oxoid, Johannesburg, South Africa) and aerobically incubated at 30 °C for 24 h. After incubation, 10 µL of the BB was dropped on a 0.65 µm filter paper placed on a tryptic blood agar plate (TBA) and allowed to incubate at room temperature for 30 min. The filter papers were carefully removed, and the plates were incubated at 37 °C for 24 h in MAE for the cultivation of *Campylobacter.* For the growth of *Arcobacter*, 10 µL of the AB was pipetted onto a 0.65 µm filter paper on a blood agar (BA) plate, incubated for 30 min at room temperature, and then the filter papers were carefully removed and the plates were aerobically incubated at 37 °C for 24 h [98,99].

### 4.3. Preparation of Positive Controls 

Bacterial-specific genomic DNA was used as a positive amplification control in the real-time PCR screening process. Genomic DNA was extracted from pure cultures of *C. jejuni* [American Type Culture Collection (ATCC) 33560] and *C. coli* (ATCC 33559) using the method described [99]. The diarrhoeic *E. coli* strains used in this study included EAEC (Strain 3591-87), EIEC (ATCC 43892), EPEC (strain B170) and EHEC (strain O157:H7) as previously reported [100]. These *E. coli* strains were previously biochemically confirmed in the Department of Microbiology of the University Stellenbosch, South Africa. The presence of the individual virulence genes was also confirmed by using PCR before conducting the experiments.

### 4.4. Genomic DNA Extraction and PCR Identification of Arcobacter and Campylobacter Species 

The total faecal genomic DNA was extracted from stool specimens using the ZR Faecal Microbe DNA Miniprep^TM^ kit (Zymo Research Corp., Irvine CA, USA) according to the manufacturer’s instructions and from pure culture isolates. The quantity and purity of the DNA obtained were assessed using a Nanodrop 1000 instrument (Nanodrop, San Francisco CA, USA). Extracted DNA was kept at −20 °C and used as template DNA for the real-time PCR.

Genus- and species-specific primers were used for the identification of *Campylobacter* together with the cycling conditions have previously been described [101,102]. Additionally, the identification of *Arcobacter* to the genus level and the simultaneous identification of *A. butzleri*, *A. cryaerophilus* and *A. skirrowii* was as previously described by [103].

### 4.5. Amplification of A. butzleri Virulence Gene

All confirmed *A. butzleri* samples were assessed for the presence of the *cadF* (*Campylobacter* invasion protein B) and *ciaB* genes (*Campylobacter* adhesin to fibronectin F), with primers and conditions described by Ghunaim et al. [104]. For each reaction, a total of 10 µL of SensiFAST^TM^ HRM Master Mix (Bioline GmbH, Luckenwalde, Germany), to which 0.5 µL (final concentration 0.5 µm) of each of the primer sets (Forward and reverse), 3 µL of template DNA and 1 µL of NF H_2_O was added.

### 4.6. Isolation of Escherichia coli from Stool Samples

The isolation of *E. coli* from stool samples was carried out using the Colilert-18 Quanti-tray/2000 (IDEXX Laboratories, Inc., Johannesburg, South Africa) Briefly, 2 g of stool sample was thoroughly mixed with 100 mL of distilled water in a sterile vessel, processed and incubated following the manufacturer’s instructions. After incubation at 37 °C, presumptive *E. coli* isolates were harvested from fluorescent Quanti-trays wells and streaked onto an Eosin methylene blue (EMB) agar to obtain pure colonies according to the method described by Abia et al. [105]. Purified colonies were stored at −80 °C in a 50% glycerol for further analysis.

### 4.7. DNA Extraction and Detection of E. coli Virulence Genes (VGs) from Pure Culture 

DNA was extracted from selected *E. coli* colonies by using the heat lysis method and whole stool sample using the ZR Faecal Microbe DNA Miniprep^TM^ kit (Zymo Research Corp., USA) according to the manufacturer’s instructions. Extracted DNA templates were tested for the presence of the malate dehydrogenase (*mdh*) gene, which is found in most *E. coli* strains. Isolates that harboured the *mdh gene* were tested for the presence of eight VGs—*eaeA* (EPEC/EHEC), *eagg* (EAEC), *ipaH* (EIEC), *ST* and *LT* (ETEC), *stx1*, *stx2* and *flicH7* (EHEC)—as previously described [106,107]. All the PCR assays included a positive control consisting of DNA from a reference strains obtained from the Microbiology Laboratories of the Natural Resources and the Environment (NRE) and CSIR, as well as previously characterised by Abia et al. [101]. A reaction mixture void of template DNA was also included in each PCR assay as a no template control.

### 4.8. Identification of Viral Pathogens

Stool samples were prepared by suspending 2 g of stool into 180 µL of phosphate buffered saline (PBS) and homogenized using a vortex. The total ribonucleic acid was extracted from 140 µL of the stool suspension using the QIAamp mini RNA kit (QIAGEN, Tokyo, Japan) according to the manufacturer’s instruction.

The identification of rotavirus and norovirus was performed using one-step SensiFast^TM^ SYBER No-ROX kit (Bioline GmbH, Luckenwalde, Germany) and primers published by Onori et al. [107]. The real-time PCR reaction mixture had a final reaction volume of 20 µL, consisting of 10 µL of the SensiFast^TM^ SYBER No-ROX kit, 0.8 µL of each of the primer sets (*Vp6*, and *RNApol* [Forward and reverse] final concentration, 400 nm of each primer), 0.2 µL of reverse transcriptase, 0.4 µL of RiboSafe RNase inhibitor, 3.8 µL of DEPC H_2_O, and 4 µL of the template DNA.

A Corbett Life Science Rotor-Gene^TM^ 6000 Cycler (Qiagen, Hilden, Germany) was used for the PCR assays under the following conditions: reverse transcription at 45 °C for 10 min, polymerase activation at 95 °C for 2 min followed by 40 cycles of denaturation at 95 °C for 5 s, annealing at 60 °C for 10 s, and an extension at 72 °C for 5 s. A melt curve analysis of the amplified product was performed using the Rotor-Gene™ real-time analysis software, version 6.1 (build 93) (Corbett Life Science (Pty) Ltd., Sydney, Australia).

### 4.9. Statistical Analysis

The results were analysed using the SPSS (Statistical Package for the Social Sciences; IBM Corporation, Armonk, NY, USA) software, version 20.0. The chi-square test was used to determine the relationship between the different PCR results obtained and other parameters such as age, diarrhoeal status, and mode of feeding. The statistics were considered significant when the *p*-value was less than 0.05.

## 5. Conclusions

The current study indicates that *Campylobacter*, *Arcobacter* spp., DEC, rotavirus and norovirus are among the contributors of diarrhoea in the Madibeng District of the North-West Province of South Africa. The results suggest that culture-based methods and a PCR assay should be used simultaneously in the laboratory for the diagnosis of diarrhoea to avoid missing some aetiologic agents, as this could adversely affect treatment outcome. The results also indicated the prevalence of mixed pathogens among children in rural areas and emphasised the need to develop approaches to examine mixed aetiology and to think beyond the one-organism one-disease concept. In addition to the investigated organisms, other bacterial pathogens that are not routinely screened for in the hospital setting may be responsible for a number of episodes of diarrhoea in babies. The prevalence of mixed pathogens in heathy individuals also prove that healthy children can act as carriers of virulent pathogens. There is, therefore, a need for the constant monitoring of aetiologic agents of diarrhoea in children. Knowledge of these agents is needed to set off future research involving inter-microbial interaction, as this could assist in defining the environmental factors that contribute to the mixed aetiology of diarrhoea infections within the same host.

## Figures and Tables

**Table 1:** Distribution of study participants according to age, gender, feeding regime and clinical symptoms.

Age (Months)	Total	Gender		Exclusive Breastfeeding	Mixed Feeding	Diarrhoea	Non-Diarrhoea	Bloody Diarrhoea	Vomiting	Fever
		Male	Female							
0–12	414	206	208	184	230	266	148	61	122	167
13–24	81	48	33	0	81	75	6	19	32	37
25–36	9	3	6	0	9	8	1	1	4	4
37–48	1	0	1	0	1	1	0	1	1	1
Sub total	505	257	248	184	321	350	155	82	159	209

**Table 2:** Distribution of aetiologic agents according to clinical symptoms *.

Aetiologic agent (n)	Diarrhoea (%)	Fever (%)	Vomiting (%)	Bloody Diarrhoea (%)
***Campylobacter*** (274)	152 (55.5)	150 (54.7)	109 (39.7)	32 (11.6)
***E. coli* (235)**	151 (64.2)	99 (42)	95 (40.4)	36 (15.3)
**Rotavirus (118)**	68 (57.6)	47 (39.8)	35 (29.6)	0
**Norovirus (101)**	77 (76.2)	55 (54.4)	40 (39.6)	0
***Arcobacter* (80)**	56 (70)	46 (57.7)	37 (64.2)	20 (25)

* Percentages are relative to the positive samples.

**Table 3:** Distribution of DEC in symptomatic and asymptomatic children according to feeding regime.

Parameters	*Stx1*	*flicH*	*eaeA*	*eagg*	*ipaH*	*Stx2*	*ST*	*LT*
Exclusive breastfeeding	2	4	7	6	3	9	6	0
mix-feeding	2	10	27	36	6	20	14	5
Diarrhoea	15	20	22	32	24	22	17	6
Non-diarrhoea	2	8	11	10	3	7	3	3
Bloody diarrhoea	6	6	8	1	6	3	4	2
Fever	13	13	13	19	18	12	11	6
No-fever	0	11	20	23	4	17	9	3
Vomiting	12	14	20	15	17	9	11	6
No-vomiting	0	10	23	27	5	20	9	3
